# Altered Thiol-Disulfide Homeostasis in Sarcoidosis: A Case–Control Study

**DOI:** 10.3390/medicina62071404

**Published:** 2026-07-20

**Authors:** Esma Andac Uzdogan, Zeynep Hande Kocaer, Salim Neselioglu, Ayşegul Karalezli, Ozcan Erel

**Affiliations:** 1 Department of Medical Biochemistry, Ankara Bilkent City Hospital, 06800 Ankara, Turkey; 2 Department of Pulmonary Disease, Bingol State Hospital, 12000 Bingol, Turkey; 3 Faculty of Medicine, Ankara Yildirim Beyazit University, 06800 Ankara, Turkey; 4 Department of Pulmonary Disease, Ankara Bilkent City Hospital, 06800 Ankara, Turkey

**Keywords:** sarcoidosis, thiol-disulfide homeostasis, oxidative stress

## Abstract

*Background and Objectives*: Sarcoidosis is a multisystem inflammatory disorder associated with significant oxidative stress. This study aimed to evaluate dynamic thiol-disulfide homeostasis (TDH)-a systemic redox status indicator-in patients with sarcoidosis. *Materials and Methods*: This cross-sectional study enrolled 58 sarcoidosis patients (diagnosed via ATS/ERS/WASOG criteria) and 53 healthy controls. Concentrations of native thiol (NT) and total thiol (TT) were determined utilizing an automated spectrophotometric technique. Disulfide (DS) levels and proportional indices were calculated, and the association of TDH parameters with the presence of sarcoidosis (patients vs. healthy controls) was examined using receiver operating characteristic (ROC) and *Cohen’s d* analysis. *Results*: Patients exhibited significantly reduced serum NT and TT levels compared to the control group (*p* = 0.003 and *p* = 0.009, respectively), whereas DS and the DS/NT ratio were elevated (*p* = 0.011 and *p* < 0.001). All parameters showed medium effect sizes. ROC analysis indicated that TDH parameters demonstrate an association with the presence of sarcoidosis, with AUC values ranging from 0.625 to 0.664. Among these, the DS/NT ratio showed the highest observed association with the disease group (AUC = 0.664), yielding 75.8% sensitivity and 60.3% specificity at a 5.7% cut-off. *Conclusions*: Our findings indicate that TDH is significantly altered in sarcoidosis, reflecting a systemic shift toward an oxidative state. These parameters appear to be exploratory biochemical indicators of systemic redox imbalance. To better understand their involvement in disease pathophysiology, future large-scale, prospective clinical trials might provide further insights.

## 1. Introduction

Characterized by the development of non-caseating granulomas, sarcoidosis is recognized as a multi-systemic inflammatory condition [1]. It has a global distribution across all genders and ethnic groups, with an incidence ranging from 1 to 40 per 100,000 individuals [2,3,4,5]. The disease most commonly affects women around the age of 50 [5]. Pulmonary involvement is observed in approximately 90% of cases, whereas extrapulmonary manifestations-including ocular, cutaneous, hepatic, splenic, lymphatic, upper respiratory tract, cardiac, bone and neurological involvement-occur in 10–30% of patients [1,6]. Clinically, sarcoidosis is characterized by heterogeneous manifestations including dry cough, ocular and cutaneous involvement, weight loss, fatigue, night sweats, and erythema nodosum. The disease exhibits a relapsing-remitting course with alternating periods of exacerbation and remission. Clinical outcomes range from spontaneous resolution to progressive, potentially life-threatening disease [7].

Although the etiology of sarcoidosis remains incompletely understood, accumulating evidence indicates that a complex interplay between genetic susceptibility, environmental exposures, antigenic triggers, and dysregulated immune responses contributes to its pathogenesis [8]. Furthermore, existing research suggests that the immune response might be triggered when antigen-presenting cells, specifically macrophages and dendritic cells, present a yet-to-be-identified antigen to CD4+ T cells [9,10]. Despite recent advances in research, a standardized diagnostic biomarker for sarcoidosis has not yet been established [11,12,13]. Given the chronic inflammatory nature of sarcoidosis, mounting evidence suggests that macrophage activation promotes the generation of reactive oxygen species (ROS), thereby contributing to tissue injury; nonetheless, the precise underlying mechanisms are not yet fully understood [14,15,16]. An increase in ROS levels, together with a reduction in antioxidant capacity, leads to the development of oxidative stress. Antioxidants that neutralize the effects of ROS exert their functions through both enzymatic and non-enzymatic mechanisms. Sulfur-containing (thiol; -SH) organic compounds, such as glutathione (GSH), are non-enzymatic antioxidant compounds [17,18,19,20]. Thiols are highly abundant serum molecules that are particularly susceptible to oxidation. The oxidation of thiol groups under oxidative stress conditions is a reversible process that leads to the generation of disulfide bonds, which can subsequently be reduced back to thiols by antioxidant systems, thereby maintaining dynamic thiol-disulfide homeostasis (TDH) [21]. Dynamic TDH reflects the balance between oxidative and reductive processes in proteins and serves as an indicator of systemic redox status [22]. Glutaredoxins are crucial compounds with thiol-disulphide oxidoreductase capacity that account for a substantial portion of antioxidant defense mechanisms in the lung [23]. A previous study demonstrated that the expression of this enzyme is significantly decreased in the alveolar macrophages of patients with sarcoidosis compared to the control group [24]. In the alveolar microenvironment of patients with sarcoidosis, footprints of persistent oxidative stress have been well documented despite an unchanged pool of GSH, a major low-molecular-weight thiol. However, while localized pulmonary oxidative stress has been evaluated, the comprehensive systemic status of dynamic TDH in sarcoidosis remains poorly characterized [25].

Current literature, to our knowledge, lacks any prior reports examining the association between sarcoidosis and TDH parameters. Accordingly, the primary aim of this study was to compare systemic TDH parameters between patients with sarcoidosis and healthy controls. Furthermore, we investigated variations in TDH parameters according to the pattern of organ involvement (pulmonary versus extrapulmonary). By characterizing systemic TDH alterations in sarcoidosis, we suggest that dynamic TDH parameters may reflect the systemic oxidative burden, providing an adjunctive biochemical perspective on the disease.

## 2. Materials and Methods

### 2.1. Study Design and Ethical Approval

This case–control study was approved by the Medical Research Scientific and Ethical Review Board of Ankara Bilkent City Hospital (Dated 13 May 2026, approval number: TABED 2-26-2199) and conducted in accordance with the Declaration of Helsinki.

### 2.2. Study Population and Participant Selection

The study included a total of 111 volunteers, consisting of patients aged ≥18 years attending the Pulmonary Outpatient Clinic of Ankara Bilkent City Hospital and healthy controls. A diagnosis of sarcoidosis was confirmed in 58 patients through a comprehensive evaluation of clinical, imaging, histopathological, and biochemical findings, adhering to the joint guidelines of the American Thoracic Society (ATS)/European Respiratory Society (ERS)/World Association of Sarcoidosis and Other Granulomatous Disorders (WASOG). Furthermore, a control group consisting of 53 healthy, age- and sex-matched volunteers was established. Exclusion criteria were age <18 years, pregnancy or lactation, active infection, malignancy, obesity, other inflammatory diseases, and current use of antioxidant supplementation. Before participating in the study, all subjects provided written informed consent.

Data regarding patient demographics (age, gender, smoking status) and baseline pulmonary function parameters, were retrieved to establish a complete clinical profile of the study participants.

Patients were categorized into subgroups based on their therapeutic regimens: treatment-naïve, corticosteroid (CS) monotherapy, CS combined with azathioprine (AZA), CS combined with methotrexate (MTX), CS combined with monoclonal antibodies (mAb), and hydroxychloroquine (HCQ) monotherapy.

### 2.3. Sample Collection and Preparation

All participants underwent a 12 h overnight fast prior to venous blood collection from the antecubital region for the assessment of both biochemical and TDH parameters. The samples were processed by centrifugation at 1500× *g* for 10 min, and the resulting serum was partitioned into Eppendorf tubes and preserved at −80 °C until the time of analysis.

Peripheral whole blood specimens were drawn into tubes supplemented with ethylenediaminetetraacetic acid (EDTA) to facilitate hematological analysis.

### 2.4. Laboratory Measurements and Instrumental Analysis

Routine biochemical parameters-including albumin (g/L), total protein (g/L), urea (mg/dL), creatinine (mg/dL), calcium (mg/dL), alanine aminotransferase (ALT, U/L), aspartate aminotransferase (AST, U/L), alkaline phosphatase (ALP, U/L), and C-reactive protein (CRP, mg/L)-were quantified utilizing standard photometric and colorimetric assays on the Atellica CH 930 automated clinical chemistry analyzer (Siemens Healthcare Diagnostics Inc., Tarrytown, NY, USA). Serum electrolytes, specifically sodium (mEq/L) and potassium (mEq/L), were measured on the same analyzer using integrated indirect ion-selective electrode (ISE) methodology in strict accordance with the manufacturer’s analytical protocols. Additionally, serum angiotensin-converting enzyme (ACE, U/L) activity, an established marker of disease activity [26], was determined kinetically using a direct spectrophotometric assay (BioSystems S.A., Barcelona, Spain) on a Beckman Coulter AU5800 clinical chemistry analyzer (Beckman Coulter, Inc., Brea, CA, USA). ACE values above 63.9 U/L were defined as elevated.

The white blood cell (WBC) counts, expressed in 10^9^/L, were assessed via a Sysmex XN 1000 automated hematology system (Sysmex Corporation, Kobe, Japan), a device that utilizes the technological mechanisms of fluorescence flow cytometry alongside direct current impedance.

We employed the automated spectrophotometric technique described by Erel and Neselioglu, which utilizes a modified Ellman’s reagent, to assess dynamic TDH parameters [21]. NT and TT levels were measured simultaneously, providing a direct assessment of [-SH] and [-SH] + [-S-S-] groups, respectively. In the initial phase, sodium borohydride (NaBH4) was employed to reduce dynamic disulfide (DS) bonds into free thiol groups. To ensure that neither the residual NaBH4 nor the newly generated DS bonds underwent further reduction of 5,5′-dithiobis-2-nitrobenzoic acid (DTNB), the remaining NaBH4 was neutralized using formaldehyde. Final concentrations of both NT and TT were subsequently quantified utilizing an adapted version of Ellman’s reagent. DS levels and related ratios-including disulfide/native thiol (DS/NT), disulfide/total thiol (DS/TT), and native thiol/total thiol (NT/TT)-were calculated from NT and TT values using the following formulas:

Disulfide (DS) Level=[Total Thiol (TT)−Native Thiol (NT)]/2

Disulfide/Native Thiol (DS/NT) % Ratio=(DS/NT)×100

Disulfide/Total Thiol (DS/TT) % Ratio=(DS/TT)×100

Native Thiol/Total Thiol (NT/TT) % Ratio=(NT/TT)×100

NT, TT, and DS levels are presented in µmol/L; DS/NT, DS/TT, and NT/TT ratios are presented as percentages (%). All measurements were performed using a Siemens ADVIA 1800 clinical chemistry analyzer (Siemens Healthcare Diagnostics, Tarrytown, NY, USA).

### 2.5. Statistical Analysis

Data processing and statistical evaluation were carried out employing IBM SPSS Statistics software (version 27.0, IBM Corp., Armonk, NY, USA). Based on an effect size of approximately 0.552, a power analysis (two-sided *α* = 0.05, 80% power) indicated a minimum sample requirement of 55 sarcoidosis patients and 50 healthy controls. Data distribution normality was verified via Kolmogorov–Smirnov and Shapiro–Wilk tests. Continuous variables are presented as mean ± standard deviation (SD) for normally distributed data, or as median (interquartile range, (IQR)) for non-normally distributed data, whereas categorical variables are summarized as frequencies and percentages (*n*, %). Group comparisons utilized Pearson’s Chi-square or Fisher’s exact tests for categorical data; for continuous variables, independent samples *t*-tests and Mann–Whitney U tests were applied based on normality assessments. The ability of TDH parameters to distinguish between sarcoidosis patients and healthy controls was assessed via Receiver Operating Characteristic (ROC) curve analysis, identifying optimal cut-off values through area under the curve (AUC) metrics and the Youden index. *Cohen’s d* was utilized to quantify effect sizes, and a *p*-value < 0.05 was considered the threshold for statistical significance, with all findings reported alongside 95% confidence intervals.

## 3. Results

A total of 111 individuals were enrolled in this study, which comprised a patient group of 58 subjects with sarcoidosis and a control group of 53 healthy volunteers. Table 1 summarizes the demographic characteristics of sarcoidosis patients and healthy controls.

Table 2 summarizes the percentage distributions of radiological stages, treatment modalities, pulmonary function test (PFT) results, organ involvement, and ACE levels for the sarcoidosis cohort, whereas Table 3 details the comparison of biochemical parameters between the patients with sarcoidosis and the healthy control group.

Subgroup analysis based on ACE levels (ACE > 63.9 U/L vs. ≤63.9 U/L) revealed no statistically significant differences in TDH parameters between the two subgroups (all *p* > 0.05).

Stratification of patients according to their treatment status (receiving vs. not receiving treatment) revealed no meaningful variations across any of the TDH parameters (all *p* > 0.05).

Regarding comorbidities, 47 patients (81.03%) had no chronic conditions. Among the remaining patients, 4 (6.89%) had one comorbid condition, 5 (8.62%) had two, and 2 (3.45%) had three comorbid conditions.

The most common thoracic CT finding was mediastinal lymphadenopathy > 10 mm accompanied by multiple nodules in 32 (55.2%). This was followed by isolated mediastinal lymphadenopathy > 10 mm 12 (20.7%), mediastinal lymphadenopathy > 10 mm accompanied by parenchymal infiltration 7 (12.1%), mediastinal lymphadenopathy > 10 mm accompanied by fibrosis 5 (8.6%), mediastinal lymphadenopathy > 10 mm accompanied by the Galaxy sign 1 (1.7%), and normal lung parenchyma 1 (1.7%).

**Figure 1:**
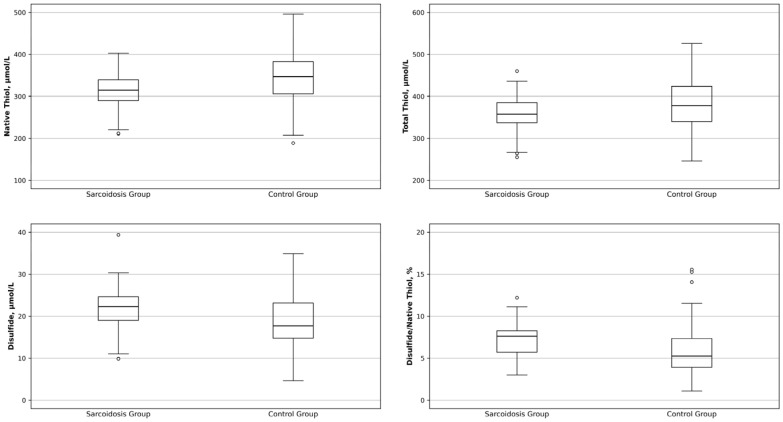
Graphical representation of NT, TT, DS levels, and the DS/NT ratio in patients with sarcoidosis and control groups. **°** represent outlier values.

Figure 1 illustrates NT, TT, DS levels, and the DS/NT ratio in both groups, while Table 4 summarizes the TDH parameters.

Analysis using *Cohen’s d* indicated statistically significant variances between the patient and control cohorts regarding all TDH parameters, demonstrating effect sizes consistently within the medium range (Table 4).

A comparison of TDH parameters in sarcoidosis patients with and without extrapulmonary involvement is presented in Table 5.

The results of the ROC curve analysis for TDH parameters are summarized in Table 6.

**Figure 2:**
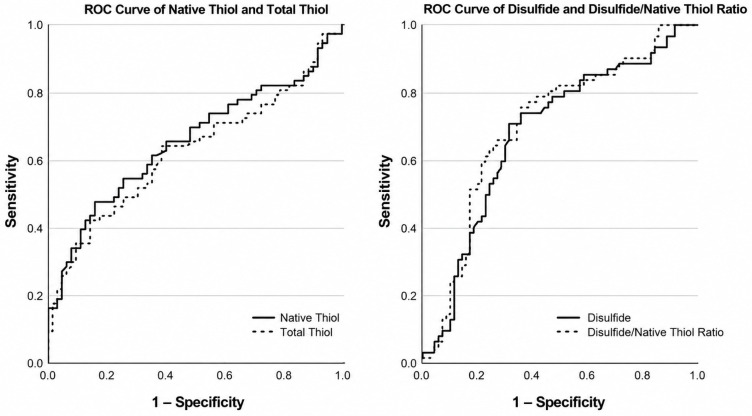
Evaluation of TDH parameters through ROC curve analysis. AUC, area under the curve; CI, Confidence Interval.

ROC curve analysis demonstrated the discriminative ability of NT, TT, DS, and the DS/NT ratio in differentiating patients with sarcoidosis from healthy controls (Figure 2).

## 4. Discussion

TDH is of vital importance and varies in response to environmental factors, disease processes, and various other conditions [17]. As far as we are aware, no previous study has investigated TDH in patients with sarcoidosis, a rare chronic granulomatous lung condition. In this study, serum NT and TT levels, which have antioxidant properties, were found to be lower in the sarcoidosis group compared with the control group. In contrast, calculated oxidant markers, including DS levels and the DS/NT ratio, were higher in patients with sarcoidosis than in controls. Thus, it has been demonstrated that in patients with sarcoidosis, the antioxidant system becomes insufficient due to oxidative stress, resulting in a shift in TDH toward an oxidative state.

The baseline demographics of our cohort strongly align with large-scale European epidemiological data. Our patients’ mean age (48.8 ± 12.8), female predominance (37; 63.8%), and high rate of non-smokers (44; 75.9%) closely mirror established epidemiological patterns [27,28]. Importantly, the patient and control groups were meticulously matched regarding age (*p* = 0.412), gender (*p* = 0.868), and smoking status (*p* = 0.780), effectively minimizing demographic confounders in our TDH analysis. This alignment not only validates our cohort’s representativeness but also corroborates that female gender and non-smoking status are prominent features of the sarcoidosis phenotype [27,28].

The distribution of extrapulmonary organ involvement in our study-particularly cutaneous, ocular, hepatic, neurological, and renal manifestations-strikingly parallels the major European cohort findings [27]. This excellent agreement further validates the representativeness of our study population. Conversely, while the European cohort demonstrated a relatively balanced distribution of Scadding stages [27], our cohort was characterized by a predominance of Stage 2 disease together with a relatively high frequency of bone involvement (22.4%). This stage distribution is consistent with recent studies identifying Scadding Stage 2 as the most common presentation in newly diagnosed sarcoidosis cohorts [29]. The notable rate of osseous manifestation in our cohort can be directly attributed to the routine incorporation of advanced imaging modalities, specifically PET-CT, during the clinical follow-up and systemic evaluation of our patients. Strongly supporting this observation, a recent study utilizing PET-CT for systematic screening reported a bone involvement rate of 22% [30], which is remarkably consistent with our findings.

Although spirometric parameters (FEV1 and FVC) remained within normal limits in most cases (87.9% and 91.4%, respectively), reduced Diffusing capacity of the lungs for carbon monoxide (DLCO) was observed in 50% of the patients. This finding is consistent with literature suggesting that impaired diffusion capacity can occur in sarcoidosis even when ventilatory parameters are preserved, reflecting early involvement of the alveolar-capillary membrane [31]. Furthermore, the predominance of mediastinal lymphadenopathy accompanied by multiple nodules (55.2%) on thoracic CT suggests a clinical presentation characterized by granulomatous involvement in our study population [31].

Although sarcoidosis is characterized by systemic inflammation, we found no statistically significant differences in routine biochemical parameters or inflammatory markers (WBC, CRP) between sarcoidosis patients and healthy controls (*p* > 0.05). These unremarkable levels of general markers may reflect the relatively stable clinical course of our cohort, which consisted predominantly of patients with Scadding Stage 2 disease [29]. Nevertheless, the borderline significance observed in calcium levels (*p* = 0.050) is consistent with the known effects of granulomatous inflammation on calcium metabolism in sarcoidosis [32]. These findings support that our patient group exhibits a phenotype characterized by granulomatous activity, while also indicating that our biochemical data should be evaluated within a broader clinical context [29].

The lack of significant differences in TDH parameters between treated and untreated patients in our cohort suggests that the administered treatment regimens do not appear to exert a substantial influence on these parameters (For all TDH parameters *p* > 0.05). Consistent with this, the absence of significant differences in TDH parameters when stratified by ACE levels indicates that serum ACE activity-at least within the ranges observed in our cohort-does not appear to be a primary driver of TDH alterations in sarcoidosis (For all TDH parameters *p* > 0.05). These observations imply that systemic oxidative stress in sarcoidosis may represent an independent pathophysiological process, operating distinctly from the granulomatous burden conventionally reflected by ACE levels. Furthermore, the persistence of altered TDH parameters regardless of treatment status suggests that standard pharmacological interventions may not sufficiently resolve the underlying systemic redox imbalance. However, these interpretations must be approached with caution; the cross-sectional design of our study and the relatively small sample sizes within these specific subgroups may limit the statistical power required to detect subtle clinical associations. Therefore, longitudinal studies with larger cohorts are needed to confirm these preliminary observations.

When *Cohen’s d* was applied, the moderate effect sizes observed across all TDH parameters indicated that the detected differences were not only statistically significant but also of biological relevance, suggesting a disruption in systemic redox homeostasis in sarcoidosis (Table 4).

The ROC curve analysis of TDH parameters yielded AUC values ranging from 0.625 to 0.664, indicating a modest ability to differentiate between patients with sarcoidosis and healthy controls. The highest AUC and sensitivity values were observed for the DS/NT ratio, suggesting that combined indices may more accurately reflect the systemic redox shift. These findings suggest that TDH parameters, rather than serving as standalone diagnostic biomarkers, may represent promising biochemical indicators of disease-related oxidative stress and systemic redox imbalance.

In studies measuring the levels of various oxidant and antioxidant molecules in patients with sarcoidosis, an overall increase in oxidative stress has been demonstrated in this patient group, supporting the findings of our study.

A study supporting our baseline findings demonstrated that serum thiol levels were significantly lower in sarcoidosis patients with either pulmonary or extrapulmonary involvement compared to healthy controls (Table 5) [33]. Interestingly, our subgroup analysis revealed a distinct pattern: patients with isolated pulmonary involvement exhibited significantly higher NT and TT levels than those with extrapulmonary manifestations. Furthermore, while the aforementioned study evaluated TDH unidirectionally by focusing solely on the antioxidant capacity, our research provides a more comprehensive assessment of dynamic TDH by simultaneously quantifying both its oxidant and antioxidant components.

In the literature, there is also a study reporting findings contrary to our results, in which thiol group levels were found to be higher in the sarcoidosis patient group compared with the control group. This finding was attributed to the possibility that, despite lower total protein levels carrying thiol groups in the patient group, homocysteine levels might be elevated in these patients; however, homocysteine could not be measured in that study. In the same study, oxidant markers including malondialdehyde (MDA), O_2_^•−^, and total oxidant status (TOS) were found to be higher in the sarcoidosis patient group compared with controls, whereas antioxidant parameters such as SOD and total antioxidant status (TAOS) were lower in the patient group [34].

In a study evaluating oxidative status at a local level, N-acetylcysteine (NAC), a thiol-containing mucolytic agent, was administered to sarcoidosis patients. In this study, an increase in GSH levels was observed in bronchoalveolar lavage (BAL) samples [35]. Thus, the local pulmonary effect of NAC, a thiol group donor, was demonstrated by an increase in GSH levels. In our study, the lower levels of NT and TT observed in the patient group suggest that thiol-containing compounds may reflect pulmonary oxidative damage at a systemic level.

In studies investigating oxidative stress parameters in other chronic lung diseases, serum samples have primarily been analyzed, along with BAL fluid and lung epithelial cells. In these studies, which also evaluated TDH, an overall increase in oxidative stress has generally been demonstrated in patients with chronic lung diseases compared with healthy control groups. A study of silicosis patients revealed lower levels of NT, TT, and a decreased NT/TT ratio relative to healthy controls [36]. Patients with chronic obstructive pulmonary disease (COPD) exhibited lower NT and TT levels compared to their healthy counterparts in a recent study. In contrast, DS levels as well as DS/NT and DS/TT ratios were higher in the patient group [37]. In a study conducted in lung cancer, NT and TT levels were likewise found to be decreased in the cancer patient group compared to the control group [38]. Collectively, these studies demonstrate that, in chronic lung diseases, the increase in oxidative stress and the inadequacy of the antioxidant defense system can be reflected by decreased serum NT and TT levels.

In a study involving patients with SARS-CoV-2 infection, it was suggested that thiols are depleted as a result of the cytokine storm induced by severe inflammation. In addition, inflammatory markers such as procalcitonin (PCT), C-reactive protein (CRP), and ferritin have been found to be higher in COVID-19 patients compared to healthy controls, and a strong correlation has been demonstrated between disease severity and these inflammatory markers. A strong inverse correlation has been observed between thiol levels and inflammatory markers such as CRP, PCT, and ferritin. Based on these data, thiols are thought to play a key role in the inflammatory response [39].

Sarcoidosis is managed using a multidisciplinary treatment approach. In sarcoidosis, treatment commonly includes immunosuppressive agents such as corticosteroids, methotrexate, azathioprine, and mycophenolate mofetil, as well as supportive therapies aimed at reducing drug-related adverse effects and managing comorbidities [40]. In the literature, there is a study conducted in sarcoidosis patients using quercetin [41], an organic compound belonging to the flavonoid family with anti-inflammatory and antioxidant properties. In this study, GSH, TAOS, and MDA levels were measured in sarcoidosis patients receiving either quercetin supplementation or placebo. As a result of these measurements, GSH levels showed no change in the quercetin-supplemented group compared to the placebo group, whereas TAOS levels increased in the quercetin group. MDA levels also showed a decrease in the quercetin-supplemented group. In the same study, analysis of post-supplementation blood samples and samples in which cytokine synthesis was induced ex vivo using lipopolysaccharide (LPS) showed reductions in anti-inflammatory Interleukin-10 (IL-10), pro-inflammatory tumor necrosis factor-alpha (TNF-α), and IL-8 cytokine levels, as well as in the TNF-α/IL-10 and IL-8/IL-10 ratios. Due to the low dose of quercetin administered to sarcoidosis patients, it was hypothesized that the amount of GSH involved in detoxification would also be limited, and therefore no change in GSH levels was observed [42].

The association between chronic inflammation and TDH in sarcoidosis may offer preliminary insights into novel adjunctive monitoring strategies. While thiol-containing formulations (e.g., GSH, NAC, and α-lipoic acid) [43] are of scientific interest as potential therapeutic targets, their clinical utility in sarcoidosis management warrants further investigation. Collectively, these findings suggest that TDH parameters represent promising non-invasive indicators of systemic oxidative stress in sarcoidosis, although their role in longitudinal clinical follow-up remains to be clarified through prospective studies.

The present study has certain limitations that should be acknowledged. Primarily, there was an uneven distribution of patients across the radiological stages of sarcoidosis. The majority of our cohort consisted of patients in Scadding Stage 2, with a complete absence of patients in Stage 3. Consequently, we were unable to perform a comparative statistical evaluation of TDH parameters across different disease stages. Furthermore, due to the limited overall sample size, we could not adequately assess the potential alterations in TDH among patients presenting with pulmonary fibrosis (Stage 4). Future large-scale studies with more evenly distributed cohorts across all radiological stages are warranted to elucidate the precise relationship between fibrotic evolution, disease staging, and oxidative stress in sarcoidosis.

## 5. Conclusions

In conclusion, our study indicates that sarcoidosis is associated with a systemic redox imbalance characterized by a shift toward an oxidative state, as evidenced by lower NT and TT levels and higher DS levels compared to healthy controls. These findings suggest that TDH parameters reflect systemic oxidative stress in sarcoidosis rather than serving as diagnostic tools. Furthermore, their clinical utility for longitudinal monitoring remains to be established through future prospective, multi-center studies. Additionally, although thiol-containing compounds are of scientific interest due to their antioxidant properties, any potential role for these formulations as adjunctive therapeutic strategies in sarcoidosis management is currently speculative. Further large-scale research is essential to determine whether these parameters offer meaningful clinical or therapeutic value in the clinical management of the disease.

## Figures and Tables

**Table 1:** Comparison of demographic variables between sarcoidosis patients and healthy controls.

Parameters	Patients (*n* = 58)	Controls (*n* = 53)	*p* Value
Age (Years)	48.8 ± 12.8 *	47.0 ± 16.1 *	0.412
Gender, *n* (%)			
Female	37 (63.8)	33 (62.3)	0.868
Male	21 (36.2)	20 (37.7)	
Smoking Status, *n* (%)			
Non-smoker	44 (75.9)	39 (73.6)	0.780
Smoker	14 (24.1)	14 (26.4)	

* Age, which demonstrated a normal distribution, is presented as the mean ± standard deviation (SD). Gender and smoking status were presented as frequency (*n*) and percentage (%).

**Table 2:** Radiological and clinical characteristics of patients with sarcoidosis.

Parameters	Patients *n* (%)	Parameters	Patients *n* (%)
FEV1 (%)		Scadding Stage	
≥80	51 (87.9)	Stage 0	1 (1.7)
<80	7 (12.1)	Stage 1	12 (20.7)
FVC (%)		Stage 2	40 (69)
≥80	53 (91.4)	Stage 4	5 (8.6)
<80	5 (8.6)	Number of Organs Involved	
DLCO (%)		1 Organ	31 (53.4)
≥80	29 (50)	2 Organ	17 (29.3)
<80	29 (50)	3 Organ	8 (13.8)
ACE (U/L)		4 Organ	2 (3.5)
≥63.9	32 (55.2)	Organ Involvement	
<63.9	26 (44.8)	Bone	13 (22.4)
Treatment		Cutaneous	10 (17.2)
No Treatment	29 (50)	Ocular	5 (8.6)
CS	21 (36.2)	Splenic	4 (6.9)
CS + AZA	3 (5.2)	Hepatic	3 (5.2)
CS + MTX	3 (5.2)	Neurological	2 (3.4)
CS + mAB	1 (1.7)	Renal	2 (3.4)
HCQ	1 (1.7)	Cardiac	0 (0)

FEV1, Forced expiratory volume in one second; FVC, forced vital capacity; DLCO, diffusing capacity of the lungs for carbon monoxide; ACE, angiotensin-converting enzyme; CS, corticosteroid; AZA, azathioprine; MTX, methotrexate; mAB, monoclonal antibody, HCQ, hydroxychloroquine. All variables in the table were presented as frequency (*n*) and percentage (%).

**Table 3:** Biochemical parameters of the patients with sarcoidosis and healthy controls.

Parameter, Unit	Patients (*n* = 58)	Controls (*n* = 53)	*p* Value
Albumin, g/L	43.96 ± 5.11	43.32 ± 4.62	0.505
Total Protein, g/L	73 (7)	71 (5)	0.111
Urea, mg/dL	33 (11)	28 (10)	0.436
Creatinin, mg/dL	0.82 (0.35)	0.76 (0.34)	0.230
Sodium, mEq/L	140.17 ± 2.64	140.27 ± 2.97	0.870
Potassium, mEq/L	4.29 ± 0.39	4.27 ± 0.43	0.845
Calcium, mg/dL	9.50 (0.6)	9.05 (1.0)	0.050
ALT, U/L	29 (15)	26 (10)	0.368
AST, U/L	18 (6)	18.5 (9)	0.252
ALP, U/L	84 (35)	73 (26)	0.649
WBC, 10^9^/L	7.19 ± 2.41	7.97 ± 2.55	0.067
CRP, mg/L	4.4 (7.85)	4.2 (8.45)	0.162

ALT, Alanine Aminotransferase; AST, Aspartate Aminotransferase; ALP, Alkaline Phosphatase; WBC, White Blood Cell; CRP, C-Reactive Protein. Albumin, sodium, potassium, and WBC counts followed a normal distribution and are reported as the mean ± standard deviation (SD). Total protein, urea, creatinin, calcium, ALT, AST, ALP and CRP levels were non-normally distributed and are presented as median ± (interquartile range (IQR)).

**Table 4:** Analysis of TDH parameters in patients with sarcoidosis compared to healthy controls.

Parameter, Unit	Patients (*n* = 58)	Controls (*n* = 53)	*p* Value	*Cohen’s d*	Effect Size
NT, µmol/L	315.78 ± 43.77	344.25 ± 64.78	0.003 *	0.51	Medium
TT, µmol/L	358.91 ± 42.08	382.07 ± 59.05	0.009 *	0.46	Medium
DS, µmol/L	21.57 ± 5.30	18.91 ± 6.47	0.011 *	0.45	Medium
DS/NT Ratio, %	7.62 (2.65)	5.25 (3.58)	0.001 *	0.42	Medium
DS/TT Ratio, %	6.61 (2.04)	4.75 (2.90)	0.001 *	0.46	Medium
NT/TT Ratio, %	86.77 (4.09)	90.49 (5.81)	0.001 *	0.46	Medium

NT, native thiol; TT, total thiol; DS, disulfide. NT, TT, and DS levels showed a normal distribution and are expressed as mean ± standard deviation (SD). DS/NT, DS/TT, and NT/TT ratios were non-normally distributed and are presented as median (interquartile range (IQR)). * *p* < 0.05 is considered statistically significant.

**Table 5:** Assessment of TDH parameters in sarcoidosis patients stratified by extrapulmonary involvement.

Parameter, Unit	Pulmonary (*n* = 31)	Extrapulmonary (*n* = 27)	*p* Value
NT, µmol/L	328.90 ± 42.59	296.35 ± 38.59	0.003 *
TT, µmol/L	371.15 ± 40.15	340.79 ± 38.83	0.004 *
DS, µmol/L	21.12 ± 6.35	22.21 ± 3.16	0.431
DS/NT Ratio, %	7.34 (3.92)	7.73 (1.61)	0.184
DS/TT Ratio, %	6.40 (3.09)	6.69 (1.21)	0.184
NT/TT Ratio, %	87.19 (6.19)	86.60 (2.43)	0.184

NT, native thiol; TT, total thiol; DS, disulfide. NT, TT, and DS levels showed a normal distribution and are expressed as mean ± standard deviation (SD). DS/NT, DS/TT, and NT/TT ratios were non-normally distributed and are presented as median (interquartile range (IQR)). * *p* < 0.05 is considered statistically significant.

**Table 6:** ROC Analysis of TDH Parameters.

	AUC (95% CI)	*p* Value	Cut-Off (µmol/L)	Sensitivity (%)	Specificity (%)	Youden Index
NT	0.654 (0.56–0.74)	0.002 *	315.7	69.9	51.6	0.215
TT	0.625 (0.53–0.72)	0.012 *	357.6	65.8	50.0	0.158
DS	0.643 (0.55–0.74)	0.004 *	20.1	71.0	64.4	0.354
DS/NT Ratio	0.664 (0.57–0.76)	0.001 *	5.7 **	75.8	60.3	0.361

NT, Native Thiol; TT, Total Thiol; DS, Disulfide; DS/NT Ratio, Disulfide/Native Thiol Ratio; AUC, Area Under Curve; CI, Confidence Interval. * *p* < 0.05 is considered statistically significant. ** The cut-off value for the DS/NT ratio is expressed as a percentage (%).

## Data Availability

The data presented in this study are available on request from the corresponding author due to privacy and ethical restrictions.
